# Cultivar-dependent phenotypic and chemotypic responses of drug-type *Cannabis sativa* L. to polyploidization

**DOI:** 10.3389/fpls.2023.1233191

**Published:** 2023-08-11

**Authors:** Hocelayne Paulino Fernandes, Young Hae Choi, Klaas Vrieling, Maikel de Bresser, Bobbie Sewalt, Francesco Tonolo

**Affiliations:** ^1^ Aboveground-belowground Interaction Group, Plant Cluster, Institute of Biology, Leiden University, Leiden, Netherlands; ^2^ Natural Products Laboratory, Institute of Biology, Leiden University, Leiden, Netherlands; ^3^ F1 SeedTech, Eindhoven, Netherlands

**Keywords:** *Cannabis sativa* L., polyploidy, cannabinoids, chemical diversity, terpenes, metabolomics, biotechnology, medicinal & aromatic plants

## Abstract

*Cannabis sativa* L. is a plant with a wide range of potential medicinal applications. In recent years, polyploidy has gained attention as a potential strategy for rapidly improving *C. sativa*, which, unlike other modern crops, has not yet benefitted from this established biotechnological application. Currently, no reports on high THCA and CBDA drug-type polyploid cultivars have been published. Moreover, it still needs to be clarified if different cultivars react similarly to polyploidization. For these reasons, we set out to evaluate and compare the phenotype and chemotype of three high Δ^9^-tetrahydrocannabinolic acid (THCA) and one high cannabidiolic acid (CBDA) drug-type cultivars in their diploid, triploid and tetraploid state through agronomic and metabolomic approaches. Our observations on plant morphology revealed a significant increase in plant height and leaf size with increasing ploidy levels in a cultivar-dependent manner. In contrast, cannabinoids were negatively affected by polyploidization, with the concentration of total cannabinoids, THCA, CBDA and cannabigerolic acid (CBGA) decreasing significantly in higher ploidy levels across all four cultivars. Headspace analysis of volatiles revealed that total volatile content decreased in triploids. On the other hand, tetraploids reacted differently depending on the cultivars. Two THCA dominant cultivars showed an increase in concentrations, while in the other two cultivars, concentrations decreased. Additionally, several rare compounds not present in diploids appeared in higher ploidy levels. Moreover, in one high THCA cultivar, a couple of elite tetraploid genotypes for cannabinoid and volatile production were identified, highlighting the role of cultivar and genotypic variability as an important factor in *Cannabis sativa* L. polyploids. Overall, our observations on plant morphology align with the giga phenotype observed in polyploids of other plant species. The decrease in cannabinoids and volatiles production in triploids have relevant implications regarding their commercial use. On the other hand, this study found that tetraploidization is a suitable approach to improve *Cannabis sativa* L. medicinal potential, although the response is cultivar and genotype-dependent. This work lays the ground for further improving, evaluating and harnessing *Cannabis sativa* L. chemical diversity by the breeding, biotechnological and pharmaceutical sectors.

## Introduction

Polyploidization is a widespread phenomenon in the plant kingdom and it plays a significant role in plant evolution and speciation. Indeed, genomic doubling provides additional genetic material upon which natural selection can act. At the same time, it establishes reproductive barriers, which can limit the capacity of polyploid organisms to mate with their diploid ancestors (Wood et al., 2009; Wendel et al., 2018). Polyploidization can lead to a multitude of effects on the plants’ phenology, morphology, physiology and metabolic processes. One of the most common effects of polyploidization is the “giga” effect. Cell and nucleus volume increases compared to diploid ancestors, with cell areas being 1.5 times larger (Lavania, 2013). This can lead to an increase in organ size and, ultimately, biomass (Urwin, 2014). Other frequently observed effects in different species are changes in size and number of stomata, shoots, roots, leaves, flowers, seeds and plant height (Trojak-Goluch et al., 2021). Because of these effects, there are many examples of plant species that have improved agronomic qualities due to polyploidization. However, polyploidy does not necessarily work for the better, as documented by Kaensaksiri et al. (2011) and Xu et al. (2014), who found that for *Centella asiatica* L. and *Echinacea purpurea* L. the number of organs was reduced, and biomass did not increase. Nevertheless, polyploidization has been a powerful breeding tool for crop improvement (Sattler et al., 2016). Simmonds (1980) estimated that approximately 40% of the cultivated species are polyploids, and today, common crops such as *Vitis* spp. L. (Motosugi et al., 2002), *Actinidia chinensis.* (Hopping, 1994), *Solanum tuberosum* L. (Carputo et al., 2003) *Prunus domestica* L. (Bennett and Leitch, 2005), and *Fragaria* (Edger et al., 2020), among many others, are polyploids.

Polyploidization has also been shown to affect secondary metabolite production in several medicinal and aromatic plants (MAPs) (Iannicelli et al., 2020), including plant species of the Cannabaceae family such as *Humulus lupulus* L. (Trojak-Goluch and Skomra, 2013). Duplication of the genome can induce a series of changes, the most obvious being the increase in gene copy number. Genome duplications can also affect the genomic organization, gene regulation, transposon activation and can induce epigenetic reprogramming by modifying histones and chromatin structures (Song and Chen, 2015). The final outcome is a change in gene expression which can directly influence the metabolome, either through increased, decreased, or silenced transcription (Madani et al., 2021).

The general trend in polyploid MAPs is an increase in secondary metabolite production (Iannicelli et al., 2020), with some exceptions (Silvarolla et al., 1999; Lavania, 2005; Wohlmuth et al., 2005; Tan et al., 2017). Various studies have reported quantitative differences of diverse secondary metabolites in synthetic autotetraploids, which could be attributed to changes in gene expression. For example, genes involved in the artemisinin biosynthetic pathway were upregulated, resulting in an increased production of artemisinin in *Artemisia annua* plants (Lin et al., 2011), and a similar change was reported for morphine and related biosynthetic genes in *Papaver somniferum* L. (Mishra et al., 2010).

In light of these findings, *Cannabis sativa* L. (Cannabaceae) appears to be an interesting candidate for polyploidization as *C. sativa* has been widely cultivated due to its industrial (Karche and Singh, 2019), ornamental (Hesami et al., 2022), nutritional (Krüger et al., 2022), and broad medicinal potentials (Andre et al., 2016) and *C. sativa* is a prolific producer of secondary metabolites with at least 348 well-characterized compounds (Hanuš et al., 2016; Pollastro et al., 2018; Radwan et al., 2021) classified as cannabinoids (150), terpenoids (120), phenolics (42), flavonoids (34) and alkaloids (2).

For these reasons, lately, polyploidization has been at the center of attention in the *C. sativa* industry. In the past, due to legal restrictions, the generation and testing of *C. sativa* polyploids posed considerable challenges as permits for handling *C. sativa* material and hazardous chemicals as well as access to specialized equipment and technical knowledge were required. These hurdles delayed basic research needed to elucidate the changes brought about by polyploidization of *C. sativa* and already documented in other plant species.

To date, there are only a handful of studies assessing the polyploid state of *C. sativa.* Mansouri and Bagheri (2017) were the first to compare tetraploid to diploid *C. sativa*. They observed that the tetraploids had more total proteins, total flavonoids and starch while having reduced cellulose content compared to diploid plants. Unfortunately, samples were not decarboxylated before cannabinoid analysis and only Δ^9^-tetrahydrocannabinol (THC) and Cannabidiol (CBD) were measured therefore missing THCA and CBDA. Mansouri and Bagheri (2017) expanded their measurements in a second experiment. They found significant differences in leaf morphology, with tetraploids having shorter and broader leaves and a decrease in glandular trichome density, fiber content, height, and soluble sugars. At the same time, the amount of total proteins doubled compared to diploids. However, cannabinoids were measured as in their first publication, and no specifications were made regarding the starting plant material. Parson et al. (2019) made observations on a single balanced CBDA/THCA drug-type tetraploid genotype and its diploid background. Measurements were conducted on ten replicates of a clone and found an 8% increase in CBDA but no increase in THCA, total cannabinoids and terpenes, while limonene decreased significantly compared to diploids. Like Mansouri and Bagheri (2017), they also observed that the central leaflet width was significantly bigger in tetraploids. Kurtz et al. (2020) published a safe and efficient method using pregerminated seeds and colchicine for developing tetraploids in *C. sativa*. They successfully generated tetraploids of 5 drug-type CBDA dominant cultivars and were able to generate triploids via embryo rescue. However, they only presented results on stomata for which they observed an increased size and reduced density with increasing ploidy levels. Lastly, Crawford et al. (2021) developed triploids and tetraploids of a CBGA dominant drug-type cultivar. Although not significant (p<0.1), they observed an increase in total cannabinoid concentrations, being 8.66%, 10.18%, and 12.38% of the dry flower weight, for diploid, triploid and tetraploid, respectively. Additionally, they also observed an increase in flower weight in triploids of 23% and 26% compared to diploids in outdoor and indoor trials, respectively.

*C. sativa* is a highly variable crop (Clarke and Merlin, 2016; Small, 2018) and Parson et al. (2019), as well as Kurtz et al. (2020), showed that the response of *C. sativa* to polyploidization induction is genotype and cultivar dependent. Additionally, observations of Parsons et al. (2019) and Crawford et al. (2021) on cannabinoid concentrations hinted that polyploidization might affect secondary metabolite quantity differently, likely dependent on the starting plant material. However, up to now, no tests on THCA and CBDA dominant drug-type varieties have been published, and it still needs to be clarified how different cultivars are affected by polyploidization. Therefore, we use a targeted metabolomics approach to investigate changes in cannabinoids and volatile content in triploids and tetraploids of 4 drug-type cultivars compared to their diploid ancestors. The cultivars used in the study, to fill the gaps in the field, were THCA and CBDA dominant and varied in secondary metabolite composition. Additionally, as leaf morphology has been proposed as proxy for polyploidy identification, we also measured leaf characteristics to check if previous observations apply to different cultivars. Here we set out to answer the following research questions: 1) Does ploidy level affect leaf morphology, cannabinoid and volatile quantity and diversity in *C. sativa*? 2) Is the response to polyploidization concerning leaf morphology, cannabinoid and volatile quantity and diversity cultivar-dependent in *C. sativa*?

## Material and methods

### Plant material

*C. sativa* seeds of four feminized commercial drug-type cultivars, in their respective diploid, triploid and tetraploid states, were obtained from F1 SeedTech. A pilot experiment was conducted, and among 29 cultivars tested, four cultivars were selected for their productivity, relatively low phenotypic variability, differential genetic background and chemical composition to evaluate their polyploid status. Cultivars used in the experiment were THCA dominant (cultivars A, B and C) and CBDA dominant (cultivar D).

### Plant growth

Plant growth was carried out following standard agricultural practices for the production of *C. sativa* inflorescences, as previously described by Magagnini et al. (2018). To minimize possible variation in the treatment of individual plants, the same nutrient solution concentration and regime of fertigation was used for all plants. Additionally, plants were placed in the growth chamber following a completely randomized design. Plants were grown in an indoor growth chamber fitted with 12, 400w HPS horticultural lights (Osram, Munich, Germany), providing a light intensity of 200 ± 50 µmol/m²/s PAR at canopy level. Environmental parameters were kept constant with a temperature of 25°C during the day and 21°C during the night, while RH was set to 70%. Diploid, triploid and tetraploid seeds were sterilized with 0.05% NaClO solution for 3 minutes and were subsequently rinsed three times with sterile deionized water and placed in Petri dishes lined with a moist paper filter. After 72h germinated seeds were then transferred to Rockwool plugs (Rockwool, Roermond, NL) moistened with hydroponic solution (Dutchpro Nutrients, Almere, NL). The plant nutrient solution was prepared by adding A and B of the vegetative hydroponic stock solution (Dutchpro) to deionized water to reach an electric conductivity (EC) of 0.4 mS/cm, and then pH was adjusted to 5.8 with NaOH. After a week from germination, plantlets were transplanted into 1l Rockwool cubes (Grodan) fitted with an automated irrigation system. During the vegetative phase, plants were fertigated three times a day with circa 150 ml vegetative hydroponic solution A and B (Dutchpro), supplemented with MgSO_4_ and CaCl_2_ to a final concentration of 0.127g/l and 0.05g/l, respectively. The EC was increased gradually as the plants grew to reach a value of 1.0 mS/cm while the surplus solution released by the Rockwool cubes was drained.

After three weeks from germination, flowering was induced by a change of photoperiod to short daylight conditions (12h/12h light/darkness). Plants then received the flowering solution A and B (Dutchpro), supplemented with MgSO_4_ and CaCl_2_, as stated above, until harvest. The EC value was increased to 1.6 mS/cm as plants entered the reproductive phase between week 7 to week 12 from germination. The nutrient solution concentration was then gradually lowered to EC 0.3 mS/cm during the last 3 weeks of flowering to promote maturation and senescence. Once all stigmas turned brown, flowers ceased to grow, and leaves progressed into senescence, plants were deemed mature. At week 15 (total of 12 weeks of flowering), flowers were harvested and dried in darkness in a climate chamber at 20°C and 30% RH for 7 days. For the number of *C. sativa* plants grown per cultivar and ploidy level, see **Table 1**. Samples for each plant were taken in equal proportion from the apical, middle and lower flowers and pooled to account for possible variability in secondary metabolite accumulation throughout the plant height. As a result, a representative 1g of dried flower material per plant was obtained. Samples were stored at -20°C in sealed 10ml Falcon tubes, and before chemical analysis, samples were grounded with a mortar and pestle until a fine powder was obtained.

**Table 1:** Number of *Cannabis sativa* L. plants grown for the experiment.

Cultivar	Diploid	Triploid	Tetraploid
A	10	4	5
B	10	7	4
C	9	5	12
D	9	5	9

### Ploidy analysis

The method for ploidy analysis was carried out following a modified protocol by Zonneveld and Van Iren (2001). Nuclei isolation buffer containing 4.15g/l MOPS, 9.15 g/l MgCl_2_, 8.8 g/l TriSodium citrate, 1.55 g/l DTT, 25g/l Polyvinylpyrrolidone and 1ml/l Triton-x was prepared before the analysis and stored at 4°C. Subsequently, 100-200 mg of fresh plant material was collected from newly emerged leaves and kept on ice until analysis. The samples were prepared by chopping the leaves with a razor blade in a Petri dish containing 1 ml of buffer. The buffer containing the free nuclei was then filtered through a 20 µm Minisart nylon filter (Sartorius, Goettingen, Germany) into a 1.5 ml Eppendorf and kept on ice while the other samples were processed. After all samples were prepared, Propidium iodide was added to a final concentration of 50 µg/ml. The samples were mixed and incubated in darkness on ice for 10 minutes prior to analysis. Ploidy analysis on a flow cytometer (Milliliter Guava Easycyte, Merck, Darmstadt, Germany) was performed following analytical parameters as stated by Parson et al. (2019) and Kurtz et al. (2020). The laser at 488nm was used for excitation, and fluorescence was recorded with Yellow H linear channel at 583/26 nm. Diploid *C. sativa* plants were used as standards. Samples were measured in triplicate and all plants part of the experiment were tested twice throughout their development (during the vegetative and flowering phase) to ensure ploidy stability, including control diploids.

### Morphological measurements

Leaf and plant height measurements were carried out two weeks before the final harvest. All plants were measured on the same day. For the leaflet measurements, the central leaflet of the leaf attached to the third node counting from the apical meristem of the plant was used. The length of the central leaflet and the width at the minor axis of the leaflet ellipse was measured. Plant height was measured from the base of the stalk to the apical meristem.

### Cannabinoid analysis

Cannabinoids were extracted with 1ml of methanol from 10 mg of homogenized flower material. Sonication was carried out for 20 minutes. Subsequently, the tube was centrifuged at 13000g to pellet plant material. The supernatant was filtered through a 20 µm RC Minisart filter (Sartorius). The extract was kept in a sealed dark glass vial and stored at -20°C until analysis. Cannabinoids were quantified following a modified protocol by Gul et al. (2015) with a reversed‐phase HPLC (Agilent 1200 chromatographic system, Agilent, Folsom, CA, USA) equipped with a UV-photodiode array detector (UV-DAD) and an auto-sampler. The separation was achieved on a Luna-C18 column (Phenomenex, Utrecht, NL). The mobile phase consisted of solvent A 0.1% (v/v) formic acid in water and solvent B 0.1% (v/v) formic acid in acetonitrile. The flow rate was set at 1.2 ml/minute. Solvent B was initially set at 30%, and after 3 minutes, it was increased to 75%. At 15 minutes from the start of the program, solvent B was increased to 100% and held for 3 minutes, after which it was decreased to 30% in the final two minutes of the sample run. The auto-sampler automatically injected 5μl of the sample. Light absorption was detected by the DAD at 228, 230, 280, 320 and 360 nm. The wavelength chosen for quantification was 320 nm. Quantification was performed with standards, THCA, CBDA, CBGA, THC, CBD and Cannabinol (CBN) (Merck). A five-fold serial dilution of the standards was performed on the range of quantification, and linear regression in RStudio software (version 4.3.0) was performed. R² for all calibration regressions were above 0.99.

### Headspace analysis

Thirty milligrams of dried plant material were placed into a 20 mL headspace vial and sealed. The samples were incubated at 150°C for 30 minutes at 500 rpm before analyses. Before capping the vial, an aliquot of 15 µL of methyl palmitate (5mg/mL), used as internal standard, was added.

The GC-MS analysis was performed following a modified protocol (Shimadzu Scientific Instruments, 2016) using a 7890A gas chromatograph equipped with a 7693 automatic sampler and a 5975C single-quadrupole mass detector (Agilent). Compounds were separated on a DB-5 column (30 m x 0.25 mm, 0.25 µm film, J&W Science, Folsom, CS, USA). Helium (99.9% purity) was used as a carrier gas at a flow rate of 1.6 mL/min. The oven temperature was held at 80°C for 1 minute, then increased at 3°C/min to 150°C, and then increased to 250°C at 7°C/min and held for 3 min. The samples were injected with a 20:1 split and split flow of 8 mL/min. The injection volume was 500 µL. The ionization energy in EI mode was 70 eV, and a mass scan range of 50–550 amu. Data was processed using MassHunter (B.07, Agilent). Peak identification was done by comparing the ion spectra obtained from samples to ion spectra in the NIST V.2008 library.

### Statistical analysis

Two-way ANOVAs were performed with cultivar and ploidy levels as fixed factors and plant height, leaflet length, leaflet width and cannabinoid levels as dependent variables. When the normality of residuals and homoscedasticity assumptions were not met, data were Box-Cox transformed. Differences between all possible pairs of means were obtained by performing a Tukey’s Honest Significance Difference (HSD) test or a Dunnett *post hoc* test. Boxplots were made to visualize differences between treatments and/or cultivars.

The GC headspace chromatograms’ total peak areas (TIC) of compounds relative to that of the internal standard (methyl palmitate) were used for multivariate analysis. SIMCA-P (16.0) software (Umetrics, Umeå, Sweden) was used to perform multivariate analysis, applying univariate scaling to the data. Loading plots, as well as VIP, were set up to visualize results and predict possible biomarkers. The unsupervised method PCA, not requiring any *a priori* knowledge, was applied first to explore the dataset. Subsequently, a supervised method OPLS-DA was applied to identify differential components among the samples. The VIP (Variable Influence on Projection) is a parameter used for calculating the cumulative measure of the influence of individual X-variables on the model. VIP values larger than 1, point to the most relevant variables, and generally, VIP values below 0.5 are considered irrelevant variables (Galindo-Prieto et al., 2013). Thus, in the present analysis, we considered only VIP values > 1. A two-way ANOVA was performed with cultivar and ploidy level as fixed factors and total peak area as dependent variable. For the number of peaks and peak area of 33 compounds with the highest VIP score, a non-parametric Kruskal-Wallis test with cultivar and ploidy as factors was performed. Following a Kruskal-Wallis test per each individual cultivar with ploidy as factor was performed. *Post hoc* analysis to detect differences between all possible pairs of means was performed with a non-parametric Dunn test. The software that was used for these analyses is R version 4.3.0. Boxplots were made to visualize differences between means using R version 4.3.0.

## Results

### Morphological measurements

#### Plant height

**Figure 1:**
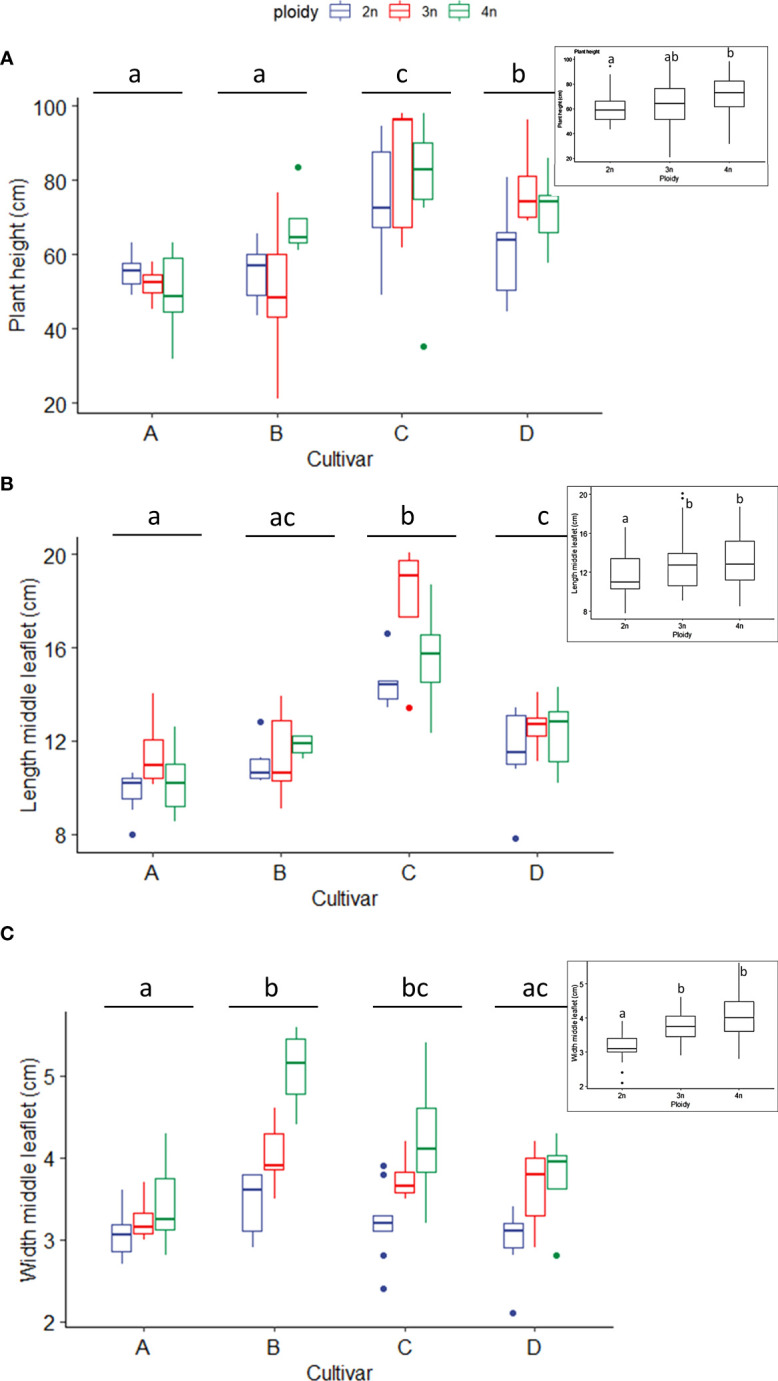
Boxplots of the **(A)** plant height (cm), **(B)** length of the middle leaflet (cm) and **(C)** width of the middle leaflet of a leaf (cm) of diploid (2n), triploid (3n) and tetraploid (4n) plants of four *Cannabis sativa* L. cultivars. **(A)**: Kruskal-Wallis rank sum test: Cultivar; χ2 = 38.269, p = 2.479e-08. Ploidy; χ2 = 7.7563, p = 0.02069. Different letters above bars indicate significant differences at p<0.05 with an all-pairs Dunn *post-hoc* analysis. The inserted graph displays the length of the middle leaflet length of the ploidy levels. **(B)** Logarithmic transformation was performed as indicated by a Box-Cox test. ANOVA: Cultivar; F_3,80_ = 46.12, p < 2e-16. Ploidy; F_2,80_ = 11.72, p = 3.42e-05. The inserted graph displays the width of the middle leaflet length of the ploidy levels. **(C)** Two-way ANOVA: Cultivar; F_3,77_ = 8.629, p = 4.98e-05. Ploidy; F_2,77_ = 27.244, p = 9.47e-10. The insert graphs show plant height between ploidy levels. Different letters above bars indicate significant differences at p<0.05 with a Tukey *post-hoc* analysis. For two samples no measurements were taken.

Measurements of plant height, as expected, revealed significant differences between cultivars. Indeed, cultivar C did have a significantly larger height than cultivars A, B and D (**Figure 1A**). Also, ploidy level did affect plant height significantly, with tetraploid *C. sativa* plants being significantly taller (71.38 ± 15.42 cm) than diploids (61.54 ± 12.99 cm). At the same time, triploids were found in-between (65.3 ± 20.77 cm). Further analysis of separate cultivars revealed a differential response to polyploidization, with cultivar A showing a decreasing trend with increasing ploidy levels. On the other hand, triploids of cultivars C and D increased compared to diploids, with D being significantly taller than its diploid ancestors. In contrast, in cultivar B a slightly significant (0.070) difference was found between the triploids, which decreased and the tetraploids, which increased compared to diploids (**Figure 1A**).

#### Leaf morphological traits

The middle leaflet length measurements also revealed significant differences between cultivars. Cultivars A and B had the shortest leaflet length, cultivar C had the longest leaflet length, while cultivar D was found in between. As expected, ploidy level also affected the leaflet length. Regardless of the cultivar, an increase was observed with increasing ploidy levels, with diploids (11.6 ± 2.0 cm) showing a significantly shorter leaflet length compared to triploids (13.04 ± 3.0 cm) and tetraploids (13.21 ± 2.6 cm) (**Figure 1B**). Both cultivar and ploidy had a significant effect on middle leaflet width. *C. sativa* cultivars B and C had significantly broader middle leaflets than cultivars A and D (**Figure 1C**). Regardless of the cultivars, the middle leaflet width increased with ploidy level, following a similar pattern observed with leaflet length. Diploids (3.17 ± 0.4 cm) showed a significantly shorter leaflet width compared to triploids (3.73 ± 0.47 cm) and tetraploids (4.09± 0.77 cm) (**Figure 1C**). Overall, leaf dimensions increased with ploidy level (**Figure S1**).

### Cannabinoids

**Figure 2:**
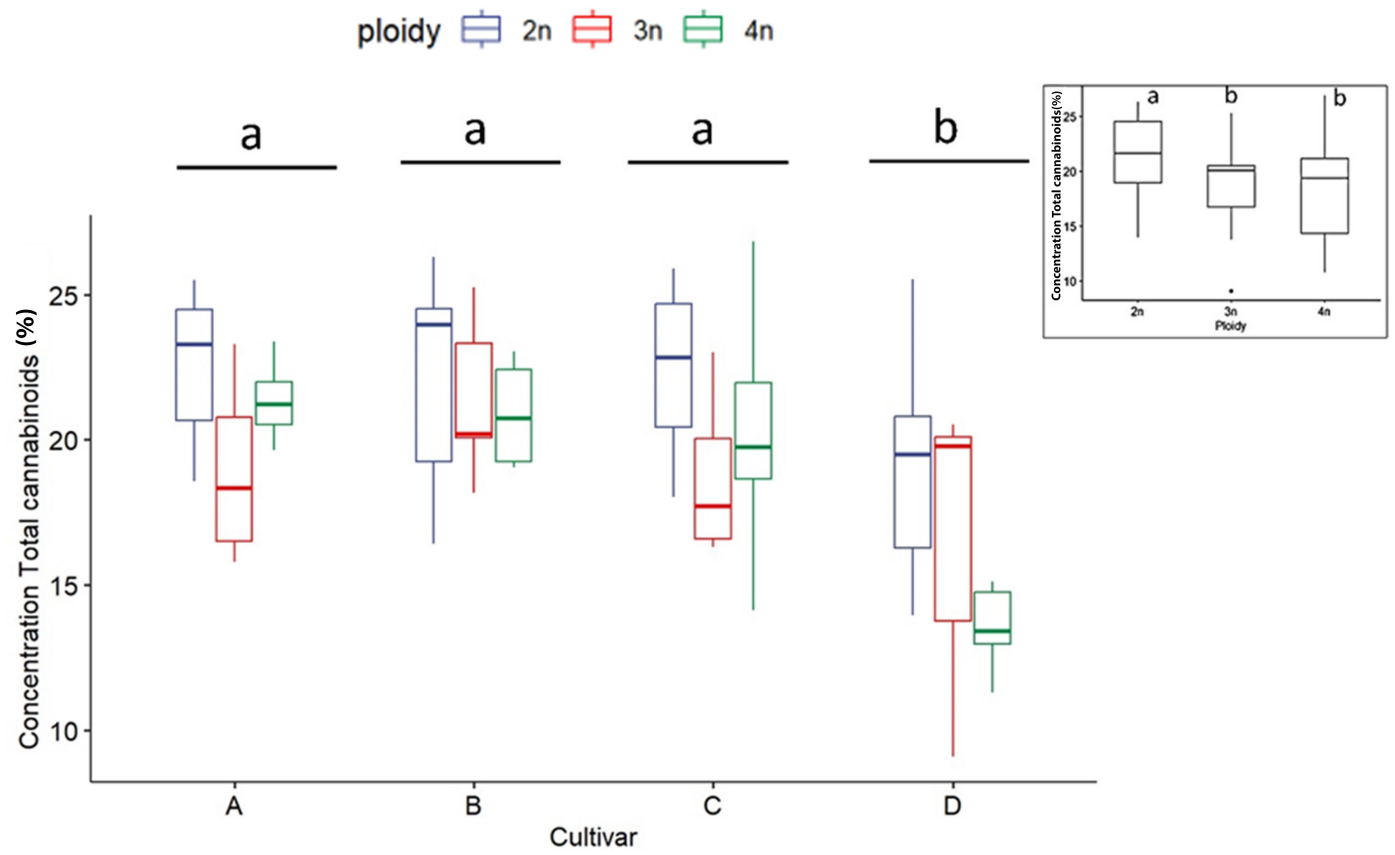
Boxplot of concentration total cannabinoids (expressed as % of dry flower weight) of a HPLC analysis of diploid (2n), triploid (3n) and tetraploid (4n) plants of four *Cannabis sativa* L. cultivars. Two-way ANOVA: Cultivar; F_3,82_ = 12.248, p = 1.06e-06. Ploidy; F_2,82_ = 8.672, p = 0.000383. The insert graph displays total cannabinoids of the ploidy levels: diploid, triploid and tetraploid. Different letters above bars indicate significant differences at p<0.05 with a Tukey *post-hoc* analysis.

**Figure 3:**
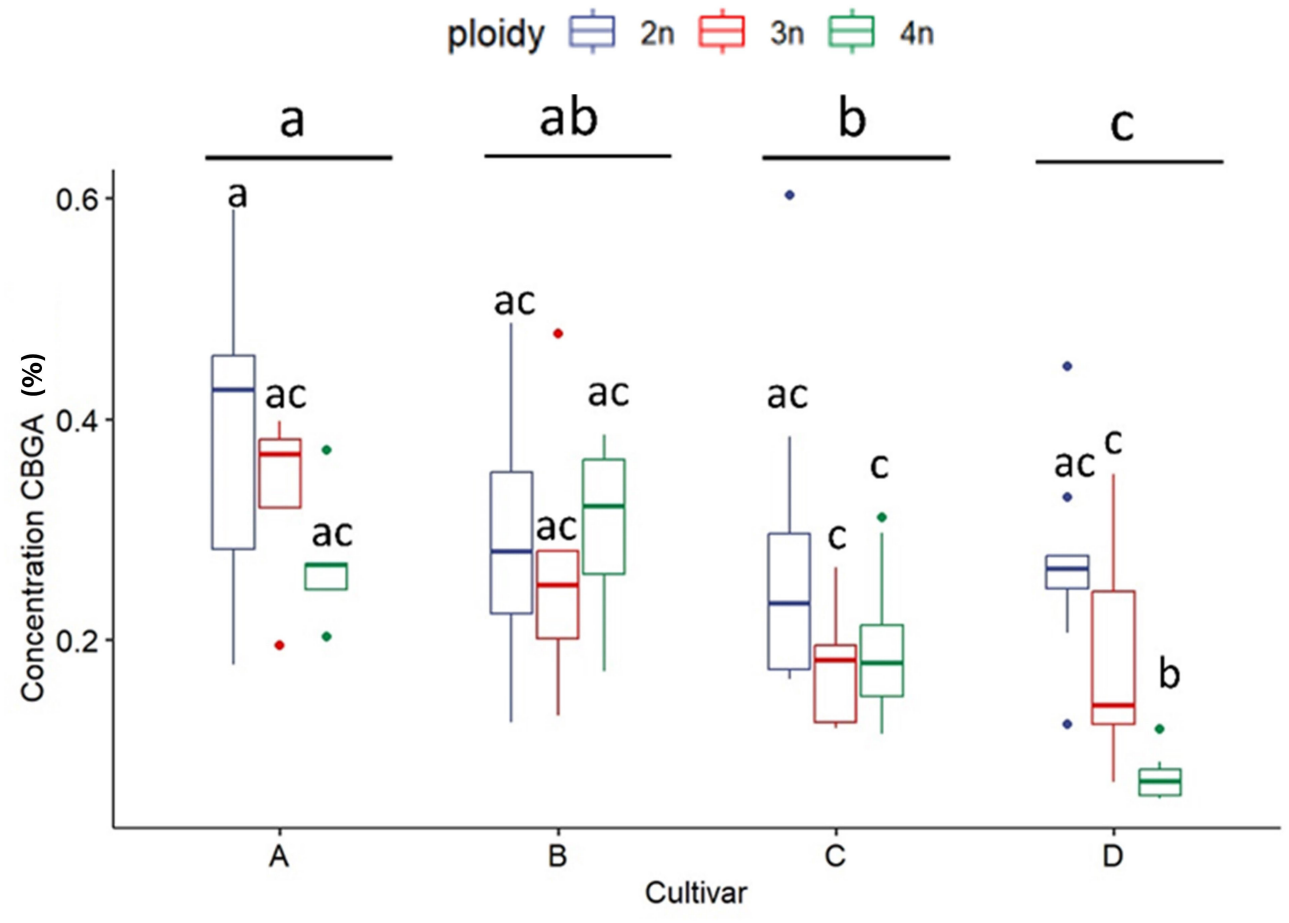
Boxplot of the concentration of CBGA (expressed as % of dry flower weight) of a HPLC analysis of flowers of diploid (2n), triploid (3n) and tetraploid (4n) plants of four *Cannabis sativa* L. cultivars. A logarithmic transformation was performed as indicated by a Box-Cox test. Two-way ANOVA: Cultivar; F_3,76_ = 15.885, p = 4.12e-08. Ploidy; F_2,76_ = 21.644, p = 3.63e-08. Cultivar x Ploidy; F_6,76_ = 4.727, p = 0.000386. Different letters above bars indicate significant differences at p<0.05 with a Tukey *post-hoc* analysis.

As expected, for all measured cannabinoid concentrations, significant differences were found between cultivars except for CBN. In all cases, CBN levels were below the threshold of detection. These findings, in combination with the detection of very low levels of THC and CBD, indicate that degradation of the cannabinoid acids during the harvesting, sample preparation and storage was minimal. The total cannabinoid levels differed significantly between cultivars, with cultivar D, the only CBDA dominant cultivar, having a significantly lower total cannabinoid content (16.47 ± 3.98%) than cultivars A (21.43 ± 2.74%), B (21.72 ± 2.80%) and C (20.64 ± 3.62%) (**Figure 2**). Total cannabinoid content was influenced by ploidy level. A decreasing trend with increasing ploidy level was observed, with total cannabinoids of diploids (21.59 ± 3.25%) being significantly higher than triploids (19.19 ± 3.73%) and tetraploids (18.51 ± 4.26%) (**Figure 2**). The precursor cannabinoid CBGA displayed differences for both cultivars and ploidy (**Figure 3**). Cultivar A had the highest level (0.34 ± 0.12%), cultivar D had the lowest level (0.18 ± 0.11%), while cultivars B (0.28 ± 0.10%) and C (0.22± 0.10%) had intermediate levels. Significant differences were detected primarily between cultivar D and the three high THCA cultivars. Similar to total cannabinoids, CBGA content was significantly affected by ploidy level, with lower concentrations associated with increasing ploidy level. Diploids had significantly higher CBGA concentration (0.31 ± 0.12%) than triploids (0.24 ± 0.11%) and tetraploids (0.18 ± 0.10%) (**Figure 3**). Moreover, the interaction was also found significant. This significant difference was driven by the very low levels in triploids and tetraploids of cultivar D and by the differential response of cultivar A, B and C. Indeed, for cultivar D, a significant -30.8% and a -71.9% decrease was detected in triploids and tetraploids, respectively. On the other hand, only a -10.5% decrease in triploids and a slight increase of +3.2% in tetraploids was detected for cultivar B. While for cultivars C and A, although a decrease was observed, it was not found to be significant, with a -37.0% and -32.2% for cultivar C, while -13.8% and -29.5% for cultivar A in the triploid and tetraploids, respectively.

As expected, the individual cannabinoids CBDA, CBD, THCA and THC significantly differed between cultivars since cultivar D is high in CBDA and very low in THCA. For this reason, a statistical analysis of THCA, THC, CBDA and CBD was performed by separating the cultivars according to the chemotype.

**Figure 4:**
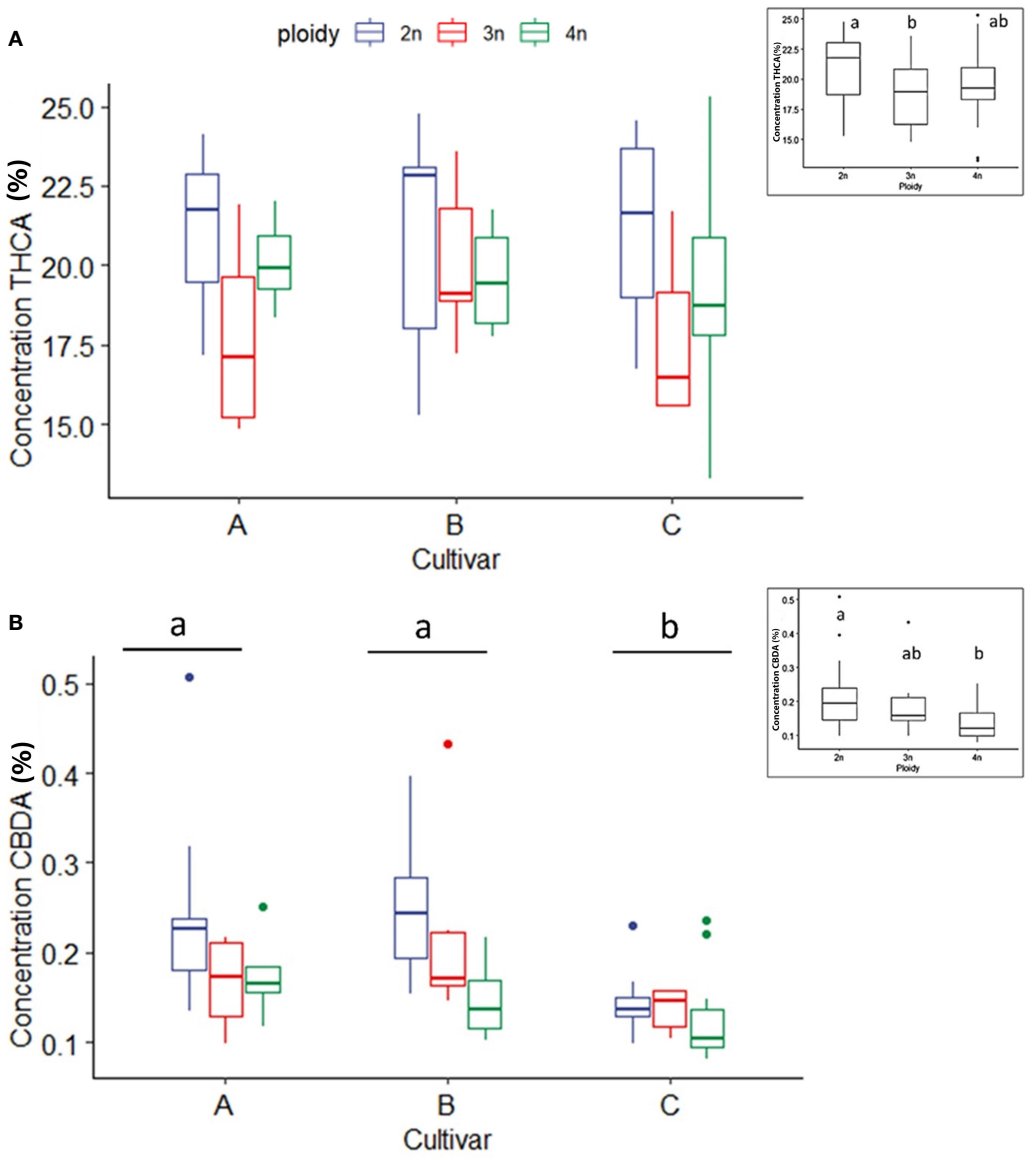
Boxplot of the concentration of THCA **(A)** and CBDA **(B)** (expressed as % of dry flower weight) of a HPLC analysis of flowers of diploid (2n), triploid (3n) and tetraploid (4n) plants of three high THCA Cannabis sativa L. cultivars. **(A)** One-way ANOVA: Ploidy; F_2,62_ = 3.452, p = 0.0379. **(B)** An inverted square root transformation was performed as indicated by a Box-Cox test. Two-way ANOVA: Cultivar; F_2,60_ = 11.127, p = 0.000196. Ploidy; F_2,60_ = 9.879, p = 0.000196. Different letters above bars indicate significant differences at p<0.05 with a Tukey *post-hoc* analysis. The insert graphs display CBDA and THCA concentration of the ploidy levels: diploid, triploid and tetraploid.

In THCA dominant cultivars (A, B and C), THCA concentrations were influenced only by ploidy levels. For all three cultivars a significant decrease in the triploids (18.8 ± 2.8%) compared to diploids (21.0 ± 2.8%) was found. Tetraploids (19.5 ± 3.0%) were not significantly different from diploids and triploids (**Figure 4A**). In the three THCA dominant cultivars, the measured CBDA content was minimal, yet an effect of polyploidization could be detected. A significant decrease with increasing ploidy level was observed. Diploids displayed the highest levels of CBDA (0.21 ± 0.09%), followed by triploids (0.18 ± 0.08%) and tetraploids (0.14 ± 0.05%) (**Figure 4B**). Analysis of THC did not reveal any differences, while CBD could not be detected for the three high THCA cultivars (data not shown).

**Figure 5:**
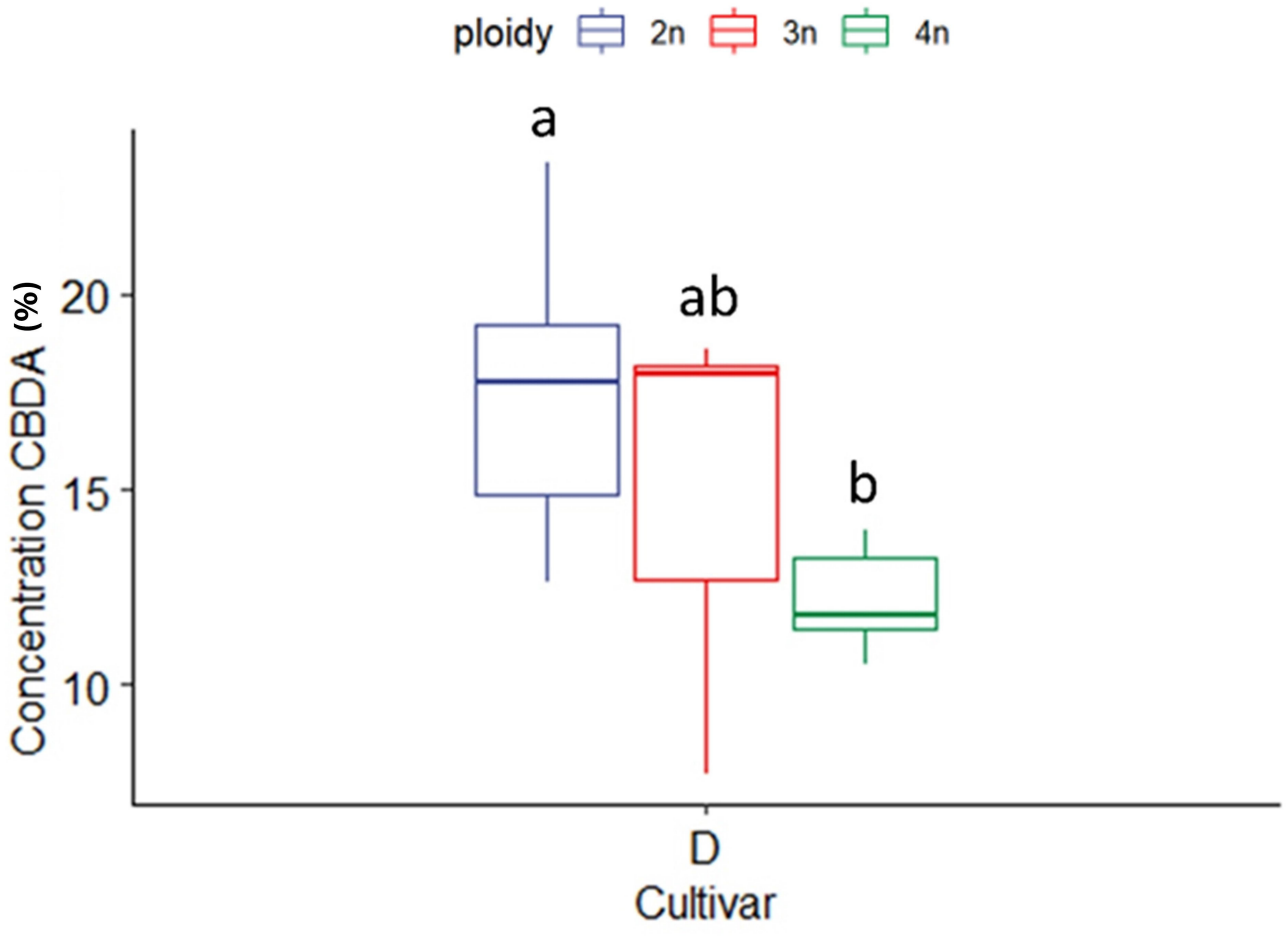
Boxplot of the concentration of CBDA (expressed as % of dry flower weight) of a HPLC analysis of flowers of diploid (2n), triploid (3n) and tetraploid (4n) plants of the high CBDA *Cannabis sativa* L. cultivar D. One-way ANOVA: Ploidy; F_2,20_ = 7.281, p = 0.004. Different letters above bars indicate significant differences at p<0.05 with a Tukey *post-hoc* analysis.

CBDA concentration in the CBDA dominant cultivar (D) was also affected significantly by ploidy level. In this case, the highest ploidy level presented the lowest CBDA concentration (12.14 ± 1.19%), while triploids had intermediate concentrations (15.02 ± 4.76%) in comparison to diploids (17.59 ± 3.20%) (**Figure 5**). For the high CBDA cultivar no differences between ploidy levels for CBD and THCA, could be found, while THC could not be detected in cultivar D.

### Headspace-GC-MS analysis

**Figure 6:**
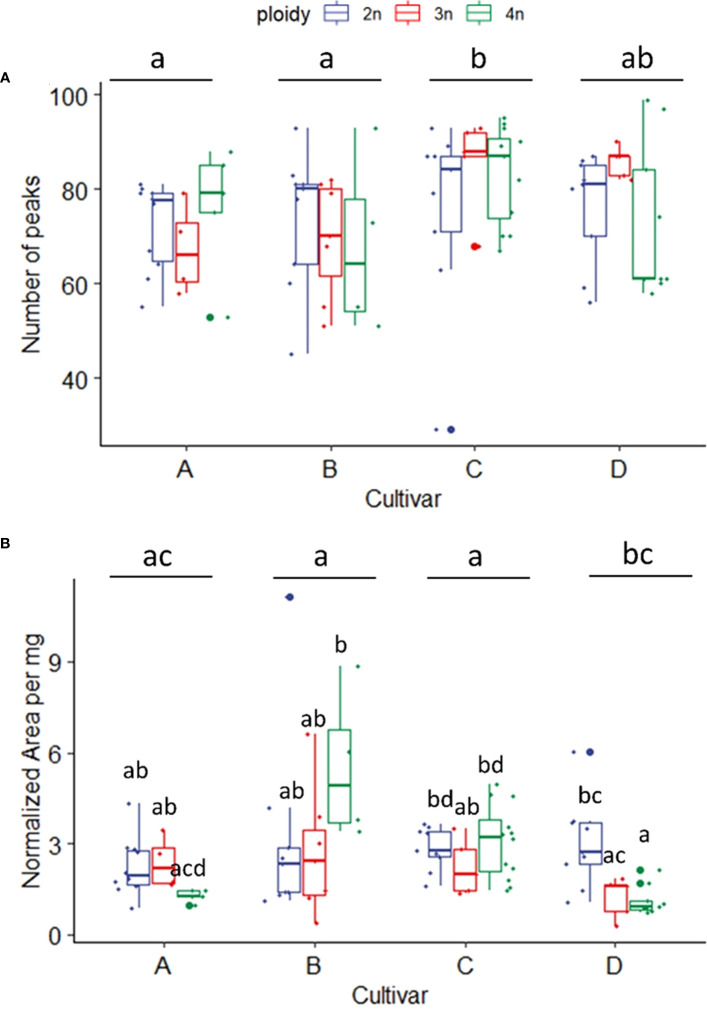
**(A)** Boxplot of total number of peaks of a headspace GC-MS analysis of diploid (2n), triploid (3n) and tetraploid (4n) plants of four *Cannabis sativa* L. cultivars. Kruskal-Wallis rank sum test: Cultivar; χ2 = 12.624, p = 0.005. Ploidy; χ2 = 0.8091, p = 0.667. Different letters above bars indicate significant differences at p<0.05 with a Kruskal-Wallis and a *post-hoc* all-pairs Dunn test. **(B)** Boxplot of total peak area per mg dry weight of a headspace GC-MS analysis of diploid, triploid and tetraploid plants of four *Cannabis sativa* L. cultivars. A logarithmic transformation was performed as indicated by a Box-Cox test. Two-way ANOVA: Cultivar; F_3,76_ = 6.558, p = 0.00053. Ploidy; F_2,76_ = 2.741, p = 0.071. Cultivar x Ploidy; F_6,76_ = 4.082, p = 0.001. Different letters above bars indicate significant differences at p<0.05 with a Tukey *post-hoc* analysis.

In total, 248 compounds were annotated (with an 85% NIST library score) from the flowers of the four *C. sativa* cultivars. As expected, most of the compounds annotated were monoterpenes (57) and sesquiterpenes (126). At the same time, also esters (2), alcohols (4), fatty acids (2), and cannabinoids such as Δ^9^-Tetrahydrocannabinol, Δ^1^-Tetrahydrocannabinol, Cannabichromene were also detected, while for the remaining 54 compounds the chemical family remained unassigned (**Table S1**). A comparison of the volatile compounds showed that cultivar C was the most diverse, containing the largest number of compounds detected (81 ± 14 peaks) compared to A (72 ± 10 peaks) and B (71 ± 14 peaks), while cultivar D was found in-between (77 ± 13 peaks). Interestingly no effect of ploidy or a ploidy x cultivar interaction was detected for number of peaks (**Figure 6A**). Cultivar B showed the highest concentration of volatiles followed by C, A and D (**Figure 6B**). The interaction between ploidy and cultivar was found significant while the effect of ploidy was detected as almost significant (p = 0.071). A decrease of -21.89% was found in the triploids compared to diploids (**Figure 6B**). While analysis of the interaction revealed a differential response of the cultivars to polyploidization regarding volatile concentrations. The main differences were detected between tetraploids of cultivar B and C, presenting the highest overall concentrations compared to tetraploids of cultivar A and tetraploids and triploids of cultivar D, which displayed the lowest overall concentrations. Additionally, a significant decrease was also detected between the diploids and tetraploids of cultivar D (-62.7%), while although not significant in cultivar B, tetraploids displayed a +75.7% increase in volatile concentrations compared to B diploids (**Figure 6B**).

**Figure 7:**
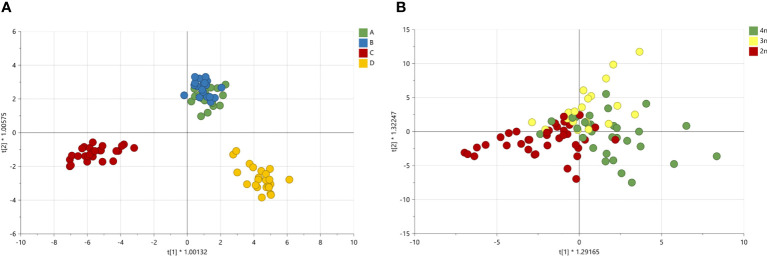
Orthogonal partial least square analysis-discriminant analysis of GC-MS analysis of 4 *Cannabis sativa* L. cultivars A, B, C and D **(A)**. The model was highly validated (R^2^X= 0.92, Q^2 = ^0.75 and p value in CV-ANOVA= 0). OPLS-DA on ploidy levels **(B)**. The model was not validated (R^2^X= 0.45, Q^2 = ^0.07 and p value in CV-ANOVA= 1).

To investigate the changes in volatiles, a principal component analysis (PCA) was carried out. The multivariate analysis showed a trend in the sample separation driven by *C. sativa* cultivars (**Figure S2A**). The main separation along the PC1 was due mainly to cultivars C and D, while the PCA plot did not show separation among the three ploidy levels (**Figure S2B**). To better discriminate cultivars and ploidy level effects, a supervised discriminant analysis, Orthogonal partial least square analysis-discriminant analysis (OPLS-DA), was used. The OPLS-DA model showed separation between the cultivars C and D while A and B clustered together (**Figure 7A**). The model was highly validated (R^2^X= 0.92, Q^2 = ^0.75 and p-value in CV-ANOVA= 0). Using the same OPLS-DA model, but differentiating the samples based on ploidy levels, a gradient separation was achieved, though the model was not validated R^2^X= 0.45, Q^2 = ^0.07 and p-value in CV-ANOVA = 1 (**Figure 7B**).

**Figure 8:**
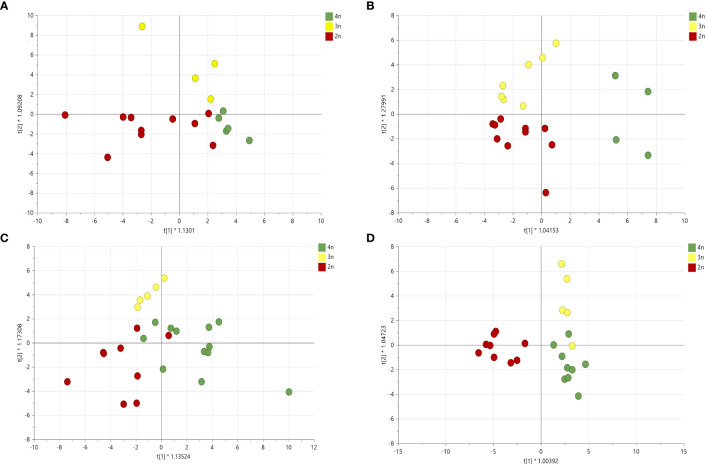
Orthogonal partial least square analysis-discriminant analysis of GC-MS analysis of four *Cannabis sativa* L. cultivars based on ploidy levels **(A)** cultivar A: Q^2^ 0.24, **(B)** cultivar B: Q^2^ 0.44, **(C)** cultivar C: Q^2^ 0.04 and **(D)** cultivar D: Q^2^ 0.27.

Given the different volatile compositions of the cultivars, we therefore also analyzed the effect of ploidy level on each cultivar separately with OPLS-DA (**Figure 8**). Although this strategy increased the resolution of ploidy separation in all four cultivars, only for cultivar B the model was validated with a Q^2^ value larger than 0.4 (**Figure 8B**).

**Figure 9:**
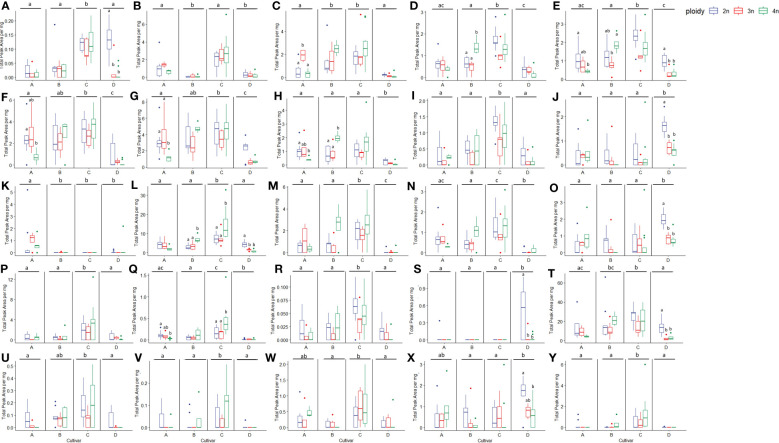
Boxplots of the total peak area per mg dry weight of 25 compounds with the highest VIP score in an OPLS-DA analysis separating cultivars of a headspace GC-MS analysis of diploid (2n), triploid (3n) and tetraploid (4n) plants of four *Cannabis sativa* L. cultivars. Kruskal-Wallis rank sum tests were performed for all compounds, for results of the tests see corresponding **Table 2**. Different letters above bars indicate significant differences at p<0.05 with a Kruskal-Wallis within the cultivar and between cultivars and *post hoc* all pairs Dunn test. **(A)** Fenchone, **(B)** Linalool, **(C)** Germacrene B, **(D)** L-α-Terpineol, **(E)** Fenchol, **(F)** γ-selinene, **(G)** Selina-3,7(11)-diene, **(H)** β-Maaliene, **(I)** *cis*-2-Norbornanol, **(J)** 3-Eudesmen-11-ol, **(K)** Butanoic acid, hexyl ester, **(L)** Caryophyllene, **(M)** Bicyclogermacrene, **(N)** β-Selinene, **(O)** Guaiol, **(P)** 1,4,7,-Cycloundecatriene,1,5,9,9-tetramethyl-,Z,Z,Z-, **(Q)** Ylangene, **(R)** 3-Methylcamphenilol, **(S)** β-Bisabolene, **(T)** D-Limonene, **(U)** Selina-5,11-diene, **(V)** Eudesm-7(11)-en-4-ol, **(W)** *cis*-α-Bergamotene, **(X)** γ-Eudesmol, **(Y)** (-)-Guaia-6,9-diene.

**Figure 10:**
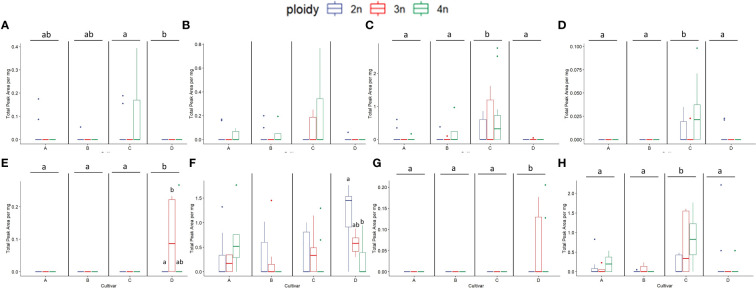
Boxplots of the total peak area per mg dry weight of 8 compounds with the highest VIP score in an OPLS-DA analysis separating ploidy levels of a headspace GC-MS analysis of diploid (2n), triploid (3n) and tetraploid (4n) plants of four *Cannabis sativa* L. cultivars. Kruskal-Wallis rank sum tests were performed for all metabolites, for their results see **Table 3**. **(A)** α-Costol, **(B)** α-Amorphene, **(C)** γ-Cardinene, **(D)** *cis*-Caryoplhyllene, **(E)** Citral, **(F)** Guai-1(10)-en-11-ol, **(G)** β-Cymene, **(H)** γ-Muurolene. Different letters above bars indicate significant differences at p<0.05 with a Kruskal-Wallis and an all-pairs Dunn test within the cultivar.

To further investigate the changes in volatiles caused by the different ploidy levels on individual compounds, 25 compounds were extracted from the model separating the cultivars. In addition, 29 compounds with a VIP score larger than one were extracted from the model separating the ploidy levels, although this model was not validated. Checking for overlapping compounds between models revealed that of the 29 extracted from the ploidy model, 21 were already present in the cultivar model. This resulted in a pool of 33 individual compounds, which except for one case (Butanoic acid, hexyl ester), concerned monoterpenes and sesquiterpenes (**Figures 9**, **10**). Further investigation revealed that changes in ploidy levels greatly influenced these individual compounds and often, cultivars were affected differently (**Tables 2**, **3**).

**Table 2:** Results of Kruskal-Wallis rank sum tests of the total peak area per mg flower dry weight of 25 compounds with the highest VIP score in an OPLS-DA analysis of a headspace GC-MS analysis of diploid, triploid and tetraploid plants of four different *Cannabis sativa* L. cultivars.

Fig	Common name	Class	Cultivar		Ploidy		Ploidy levels per cultivar							
							A		B		C		D	
			*χ2*	*p*	*χ2*	*p*	*χ2*	*p*	*χ2*	*p*	*χ2*	*p*	*χ2*	*p*
A	Fenchone	Monoterpene	36.7	0.000	4.9	0.087	0.8	0.661	0.1	0.961	2.0	0.362	14.2	0.001
B	Linalool	Monoterpene	48.1	0.000	0.9	0.649	4.4	0.112	0.8	0.673	1.8	0.400	1.9	0.384
C	Germacrene B	Sesquiterpene	48.1	0.000	2.8	0.251	8.2	0.016	4.4	0.111	3.5	0.175	3.4	0.187
D	L-α-Terpineol	Monoterpene	47.2	0.000	1.6	0.457	2.5	0.283	9.3	0.009	5.4	0.067	4.1	0.128
E	Fenchol	Monoterpene	37.0	0.000	8.7	0.013	6.0	0.050	8.1	0.018	5.7	0.059	12.0	0.002
F	γ-selinene	Sesquiterpene	37.4	0.000	0.7	0.718	7.0	0.030	0.7	0.704	2.1	0.356	2.6	0.275
G	Selina-3,7(11)-diene	Sesquiterpene	34.7	0.000	2.6	0.268	7.8	0.020	4.8	0.093	2.7	0.264	5.3	0.070
H	β-Maaliene	Sesquiterpene	35.0	0.000	0.1	0.973	6.7	0.035	8.1	0.018	3.4	0.186	4.3	0.115
I	*cis*-2-Norbornanol	Monoterpene	30.6	0.000	6.4	0.039	0.9	0.633	3.7	0.155	4.8	0.089	3.5	0.171
J	3-Eudesmen-11-ol	Sesquiterpene	26.2	0.000	3.3	0.195	1.2	0.543	5.5	0.063	1.3	0.530	16.0	0.000
K	Butanoic acid, hexyl ester	Ester	26.9	0.000	0.3	0.841	2.7	0.256	1.9	0.395	NA	NA	1.2	0.541
L	Caryophyllene	Sesquiterpene	43.8	0.000	2.0	0.368	3.7	0.150	7.5	0.023	9.8	0.007	16.9	0.000
M	Bicyclogermacrene	Sesquiterpene	39.8	0.000	2.1	0.348	0.3	0.830	4.2	0.120	2.2	0.338	1.5	0.463
N	β-selinene	Sesquiterpene	41.6	0.000	1.0	0.589	6.2	0.044	2.6	0.275	1.5	0.475	1.4	0.495
O	Guaiol	Sesquiterpene	20.4	0.000	2.7	0.255	2.9	0.237	3.5	0.171	1.3	0.534	14.9	0.001
P	1,4,7,-Cycloundecatriene, 1,5,9,9-tetramethyl-, Z,Z,Z-	Sesquiterpene	15.9	0.001	1.3	0.509	3.2	0.204	1.7	0.437	1.8	0.399	3.1	0.217
Q	Ylangene	Sesquiterpene	39.9	0.000	1.7	0.419	6.3	0.043	0.2	0.884	8.3	0.016	1.0	0.613
R	3-Methylcamphenilol	Monoterpene	25.5	0.000	4.2	0.121	1.4	0.491	2.3	0.313	2.4	0.302	3.7	0.158
S	β-Bisabolene	Sesquiterpene	24.0	0.000	4.2	0.121	0.9	0.638	NA	NA	NA	NA	7.2	0.027
T	D-Limonene	Monoterpene	26.2	0.000	7.4	0.024	5.0	0.082	3.1	0.218	4.0	0.135	12.2	0.002
U	Selina-5,11-diene	Sesquiterpene	18.9	0.000	4.1	0.127	4.2	0.120	0.7	0.714	2.3	0.317	5.4	0.066
V	Eudesm-7(11)-en-4-ol	Sesquiterpene	19.6	0.000	4.4	0.108	1.9	0.389	3.0	0.224	2.8	0.248	9.2	0.010
W	*cis*-α-Bergamotene	Sesquiterpene	19.5	0.000	0.6	0.705	3.8	0.150	2.4	0.299	1.0	0.618	1.7	0.417
X	γ-Eudesmol	Sesquiterpene	10.3	0.016	4.4	0.108	1.9	0.389	3.0	0.224	2.8	0.248	9.2	0.010
Y	(-)-guaia-6,9-diene	Sesquiterpene	31.9	0.000	4.2	0.117	1.9	0.387	1.9	0.387	3.4	0.179	1.6	0.459

Compounds are ranked with the first compound having the highest VIP score. Letters in the first row refer to corresponding boxplots in **Figure 9**. NA indicates that the compounds were not detected in the cultivar and therefore a statistical test could not be performed.

**Table 3:** Results of Kruskal-Wallis rank sum tests of the total peak area per mg flower dry weight of 8 compounds with the highest VIP score in an OPLS-DA analysis of a headspace GC-MS analysis of diploid, triploid and tetraploid plants.

Fig	Common name	Class	Cultivar		Ploidy		Ploidy levels per cultivar							
							A		B		C		D	
			*χ2*	*p-value*	*χ2*	*p-value*	*χ2*	*p-value*	*χ2*	*p-value*	*χ2*	*p-value*	*χ2*	*p-value*
A	α-Costol	Sesquiterpene	10.5	0.015	3.7	0.154	1.900	0.387	1.222	0.543	3.192	0.203	NA	NA
B	α-Amorphene	Sesquiterpene	5.6	0.131	3.6	0.160	1.608	0.448	1.807	0.405	4.987	0.083	1.556	0.459
C	γ-Cadinene	Sesquiterpene	16.3	0.001	2.2	0.324	0.916	0.633	0.586	0.746	1.245	0.537	3.600	0.165
D	*cis*-Caryophyllene	Sesquiterpene	26.3	0.000	4.9	0.084	NA	NA	NA	NA	4.146	0.126	3.253	0.197
E	Citral	Monoterpene	11.7	0.008	6.2	0.045	NA	NA	NA	NA	NA	NA	7.196	0.027
F	Guai-1(10)-en-11-ol	Sesquiterpene	8.1	0.045	4.0	0.134	2.735	0.255	2.385	0.303	2.557	0.279	8.273	0.016
G	β-Cymene	Monoterpene	11.7	0.008	3.2	0.199	NA	NA	NA	NA	NA	NA	3.613	0.164
H	γ-Muurolene	Sesquiterpene	21.0	0.000	4.3	0.115	1.943	0.379	4.079	0.130	5.416	0.067	1.553	0.460

Compounds are ranked with the first compound having the highest VIP score. Letters in the first row refer to corresponding boxplots in **Figure 10**. NA indicates that the compounds were not detected in the cultivar and therefore a statistical test could not be performed.

Analysis of the 33 compounds in triploids revealed the same pattern observed in total volatile content but on a deeper level. In triploid plants, the majority of compounds decreased with cultivar A, showing a decrease in the concentration of 22 compounds while 8 increased compared to diploids. In cultivar B, 21 compounds decreased while 7 increased. In cultivar C, 18 compounds decreased while 11 increased and in cultivar D the concentration of 27 compounds decreased while only 5 increased compared to diploids (**Table S2**). Overall, this data indicates that although a small proportion of compounds increased in concentration, the majority decreased, with cultivar D being the most affected and cultivar C the least (**Table S2**).

An interesting trend was observed in tetraploids, with cultivars B and C showing an important difference compared to cultivars A and D. In cultivar A the concentrations of 24 compounds decreased while 6 increased compared to diploids. Cultivar D also showed a decrease in concentrations of 26 compounds and an increase in 5 compared to diploids. In contrast, in cultivar B, the concentration of only 8 compounds decreased while 18 increased. Similarly in cultivar C the concentration showed a reduction in 9 compounds while 20 increased compared to diploids (**Table S2**).

Additionally, several compounds present in diploids were no longer detected in triploids and tetraploids (**Table S2**). In cultivar A, β-bisabolene, Guaia-6,9-diene, α-Costol were not detected in both triploids and tetraploids. The compounds γ-Cadinene, Eudesm-7(11)-en-4-ol and 1,4,7,-Cycloundecatriene, 1,5,9,9-tetramethyl-, Z,Z,Z- were not detected in triploids and Selina-5,11-diene was not detected in tetraploids A. In cultivar B α-Costol was not detected in both triploids and tetraploids and Eudesm-7(11)-en-4-ol and α-Amorphene were not detected in triploids while 3-Eudesmen-11-ol, Guaiol, *cis*-α-Bergamotene, Guai-1(10)-en-11-ol and γ-Muurolene were not detected in tetraploids. In cultivar C only α-Costol disappeared in triploids. On the other hand, several compounds were no longer detected in both triploids and tetraploids of cultivar D, Eudesm-7(11)-en-4-ol, Guaia-6,9-diene, α-Amorphene and *cis*-Caryophyllene while γ-Muurolene and Butanoic acid, hexyl ester were not present in triploids and Selina-5,11-diene was not present in tetraploids (**Table S2**).

Analysis across cultivars revealed that Selina-5,11-diene was not present in both tetraploids of cultivars A and D, Eudesm-7(11)-en-4-ol was not present in triploids of A, B and D, Guaia-6,9-diene was not present in both triploids and tetraploids of A and D. α-Costol was not present in triploids of A, B, C and tetraploids A and B while α-Amorphene was not present in triploids of A, B and D.

In cultivars C and D, some compounds that were not present in diploids were detected in the higher ploidy levels. In cultivar C, α-Amorphene was present in triploids and tetraploids, in concentrations that were more than doubled in tetraploids compared to triploids. Interestingly in cultivar D Citral and β-Cymene were present in both triploids and tetraploids while γ-Cadinene only was present in triploids.

Statistical analysis revealed that, for four compounds, significant differences in ploidy level were detected when pooling the four cultivars. Three compounds (*cis*-2-Norbornanol, Fenchol, and D-Limonene) showed the same pattern with strikingly similar numbers. Indeed, a significant decrease in triploids (*cis*-2-Norbornanol -55.31%; Fenchol -43.86%; D-Limonene -47.22%) and a decrease in tetraploids (c*is*-2-Norbornanol -20.94%; Fenchol -18.91%; D-Limonene -27.54%) was observed (**Figure 9**). On the other hand, Eudesm-7(11)-en-4-ol showed an increase in tetraploids (+85.39%) while there was a decrease in triploids (-81.77%), leading to a significant difference between triploids and tetraploids plants. Further analysis on Eudesm-7(11)-en-4-ol revealed that in triploids of cultivar A, B and D, the compound was undetectable, while the increase in the tetraploids was driven by cultivar B (+111.23%) and C (+112.92%) (**Figure 9**).

Fenchone decreased in both ploidy levels across the four cultivars, apart from tetraploid C, where a slight increase of +5.45% was detected. Moreover, the decrease in cultivar D was found to be significant, with -81.52% in triploids and -91.73% in tetraploids. β-Bisabolene was undetectable in cultivars B and C, while in cultivar D, it significantly decreased in both triploids (-88.30%) and tetraploids (-94.43%) and decreased under detection level in both triploids and tetraploids of cultivar A. The remaining compounds all showed a differential response to polyploidization depending on the cultivars under analysis, particularly comparing tetraploids of cultivar A and D versus B and C (**Table S2**).

Some of the most striking differences were detected in β-Maaliene, where a significant decrease was detected in tetraploids of cultivar A (-57.67%), while for cultivar B a significant increase was detected in tetraploids (+164.82%). Ylangene also showed a differential response of the cultivars in the tetraploid state, with cultivar A showing a significant decrease of -72.75%. In contrast, a significant increase of +182.13% for cultivar C was observed. Similarly, for β-Selinene, cultivar A showed a significant decrease in tetraploids (-56.66%) while cultivar B, although not significantly, increased by +113.96% in tetraploids. For γ-Eudesmol, cultivar D showed a significant -69.06% decrease in tetraploids, while cultivar A showed a +105.39% increase. Analysis of *cis*-α-Bergamotene revealed that concentrations in tetraploids of cultivar A increased by +78.04%, in cultivar C by +83.09%, while in tetraploids of cultivar B, the compound was not detected anymore. Interestingly, the rare compound Guaia-6,9-diene increased in tetraploids of cultivar C by +145.45% and +3378.74% in tetraploids of cultivar B. On the other hand, the compound could not be detected anymore in tetraploids of cultivars A and D.

**Figure 11:**
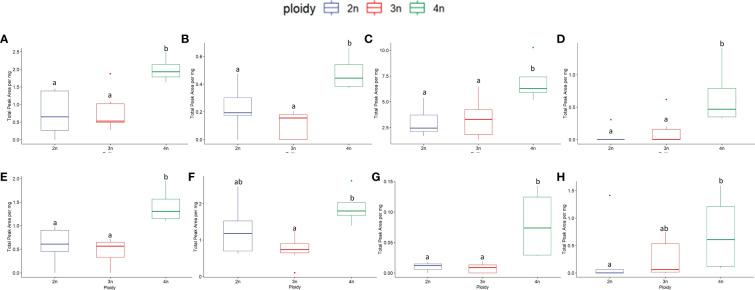
Boxplots of the total peak area per mg flower dry weight of 8 compounds with the highest VIP score in an OPLS-DA analysis separating ploidy levels of a headspace GC-MS analysis of diploid (2n), triploid (3n) and tetraploid (4n) plants of *Cannabis sativa* L. cultivar B. ANOVAs were performed to assess the differences between ploidy levels for this cultivar in the case of non-parametric data the non-parametric alternative Kruskal-Wallis rank sum tests was performed, for results see **Table 4**. Different letters above bars indicate significant differences at p<0.05 of an ANOVA and Tukey *post-hoc* analysis or a Kruskal-Wallis and an all-pairs Dunn test. **(A)** β-Maaliene, **(B)** *endo*-Borneol, **(C)** Caryophyllene, **(D)** 3-Carene, **(E)** L-α-Terpineol, **(F)** Fenchol, **(G)** β-Thujene, **(H)** *trans*-2-Pinanol.

Since cultivar B showed for several traits a positive reaction to polyploidization and the OPLS-DA model was validated, the analysis was also performed on the VIPs compounds of cultivar B separately (**Table 4**). The analysis was performed on 15 compounds, of which 8 showed a significant increase in cultivar B tetraploids compared to diploids (β-Maaliene +164.83%; Endo-Borneol +120.50%; Caryophyllene +118.4%; 3-Carene +1874.68%; L-α-Terpineol +150.87%; Fenchol +59.39%; β-Thujene +692.22%; *Trans*-2-Pinanol +309.3%) while two increased although the statistics could not detect a significant effect of ploidy (D-Guaiene +298.38%; Bicyclogermacrene +274.77%) (**Figure 11**).

**Table 4:** Results of ANOVAs or Kruskal-Wallis rank sum tests of the total peak area per mg dry flower weight of 8 compounds with the highest VIP score in an OPLS-DA analysis of a headspace GC-MS analysis of diploid, triploid and tetraploid plants of cultivar B. Letters in the first row refer to corresponding boxplots in **Figure 11**.

Fig.	Common name	Class	Ploidy		Ploidy	
			F-value	p-value	χ2	p-value
A	β -Maaliene	Sesquiterpene	8.565	0.003		
B	*endo*-Borneol	Monoterpene	10.040	0.001		
C	Caryophyllene	Sesquiterpene	8.460	0.003		
D	3-Carene	Monoterpene			10.723	0.005
E	L- α-Terpineol	Monoterpene	10.980	0.001		
F	Fenchol	Monoterpene	6.562	0.008		
G	β -Thujene	Monoterpene			9.505	0.009
H	*trans*-2-Pinanol	Monoterpene			5.776	0.056

Additionally, analysis of outliers detected an interesting effect of polyploidization in cultivar C. Several genotypes in this cultivar consistently presented very high levels of rare compounds compared to diploid ancestors, identifying them as elite tetraploid genotypes. Indeed, plant ID 82 presented a large increase in 8 compounds compared to diploid (β-Maaliene +336.89%; Caryophyllene +354.78%; 1,4,7,-Cycloundecatriene, 1,5,9,9-tetramethyl-, Z,Z,Z- +504.81%; Ylangene +830.39%; β-Selinene +145.86%; Guaia-6,9-diene +1024.29%; α-Amorphene +inf%; α-Costol +932.78%) while plant ID 91 showed a marked increase in 3 rare compounds (Guaiol + 569.55%; *cis*-Caryophyllene +913.95%; 3-Eudesmen-11-ol +527.32%). Moreover, further investigation revealed that plant ID 82 was also a top performer for total volatile content being sixth out of 89 plants. Given these results, cannabinoid data was also screened, and C tetraploid genotype ID 85 was found to be the best performer for total cannabinoids (26.84%) and linalool (+292% compared to average diploid).

## Discussion

In recent years, polyploidy has gained attention as a potential strategy for rapidly improving *C. sativa*. Compared to other modern crops, *C. sativa* has yet to benefit from this established biotechnological application. Indeed, the observed effects on plant morphology and secondary metabolite production in other medicinal and aromatic plants make it a very promising field of research.

Here we found that contrary to previous observations (Mansouri and Bagheri, 2017), plant height significantly increased with increasing ploidy levels. However, analysis of separate cultivars also highlighted a differential response, with cultivar D showing significantly taller triploids than diploids, while B triploids were shorter than their diploids counterpart. On the other hand, leaf morphology observations agreed with previous observations (Mansouri and Bagheri, 2017; Parsons et al, 2019). Leaf size increased significantly, with both leaflet length and width increasing with increasing ploidy level. Overall, our observations also align with the “giga” phenotype observed in other plant species after polyploidization (Sattler et al., 2016).

Investigation of secondary metabolites revealed several surprising changes. First, cannabinoids appear to be negatively affected by polyploidization, as the concentration of total cannabinoids decreased in higher ploidy levels. Moreover, major cannabinoids THCA, CBDA, as well as CBGA followed the same trend as observed for total cannabinoids. The significant decrease in concentration observed in total cannabinoids, -13.27% in triploids and - 28.89% in tetraploids of the CBDA dominant cultivar, and -11.76% in triploids and -6.89% in tetraploids of THCA dominant cultivars, has relevant implications regarding the uses of polyploids for producing major cannabinoids. These results are in contrast with the observation by Crawford et al. (2021), which observed an increase in total cannabinoids in polyploids of a drug-type CBGA dominant cultivar, highlighting once more the cultivar-dependent response to polyploidization. Regarding the concentration of CBGA, the results agree with the observation of Parson et al. (2019) who also detected a circa -30% decrease. Moreover, considering that the limitation on the availability of the precursor cannabinoid CBGA is a strong limiting factor for the accumulation of downstream cannabinoids (Laverty et al., 2019). These data point to the probable downregulation of the cannabinoid pathway in the higher ploidy levels. From these results, it appears that in modern drug-type *C. sativa* cultivars, where cannabinoid production has already been pushed to extremely high levels (>20% of dry flower weight) by artificial selection, in an event such as polyploidization, where plants undergo a highly stressful genomic reorganization, changes might favor other metabolic pathways as observed by Mansouri and Bagheri (2017).

A much more complex suite of changes was detected regarding the presence and concentration of volatiles. First of all, triploids showed a clear decrease in total volatile content. On the other hand, tetraploids depending on the cultivars, reacted differently, with cultivars B and C affected positively, while in cultivars A and D, concentrations decreased.

The VIPs extracted from the multivariate analysis yielded the 33 individual compounds having the most influence on the separation between cultivars and ploidy levels. Analysis of these compounds highlighted to a deeper level several interesting findings. Generally, triploids’ volatiles were negatively affected across cultivars, apart from specific cases. Plants in the tetraploid state showed a differential response, with many compounds in C and B increasing while at the same time in A and D decreasing. Interestingly, several rare compounds appeared in higher ploidy levels. Cultivar C presented a couple of elite tetraploid genotypes, highlighting the role of within cultivar genotypic variability as an important factor in *C. sativa* polyploids. In cultivars A and D, the concentrations of most of the compounds decreased, and several were not detected in higher ploidy levels, pointing to the downregulation of the terpene pathway in these cultivars. On the other hand, in cultivar B, although an increase in concentration was detected for most of the compounds at higher ploidy levels, some also disappeared. These changes point to the probable gene-silencing effects caused by polyploidization in this specific cultivar (Eckardt, 2010).

This complex suite of changes have important implications for the medicinal value of the plants. Indeed, terpenes play an essential role in the bioactivity of *C. sativa* extracts and can contribute to pharmacological activity via the entourage effect (Russo, 2011). Some compounds that were found to increase significantly in cultivars B and C display important medicinal properties. “*Endo*-Borneol can increase drug delivery across various physiological barriers (Kulkarni et al., 2021), 3-Carene has sleep-enhancing effects by targeting the GABAA-benzodiazepine receptors (Woo et al., 2019), L-α-Terpineol displays anticancer, anticonvulsant, antiulcer, antihypertensive, anti-nociceptive properties (Khaleel et al., 2018) and β-Caryophyllene might become effective for the treatment of diabetes and associated complications (Hashiesh et al., 2020). Moreover, Guaia-6,9-diene, a rare compound with promising anti-SARS-CoV-2 properties, also greatly increased (Amparo et al., 2021).

Likewise, a decrease in cultivars A and D of compounds with relevant medicinal properties would probably affect the cultivars’ medicinal potential. The concentration of some compounds which decreased in higher ploidy levels show promising antitumor activity, such as Guaiol and β-bisabolene (Yeo et al., 2016; Yang et al., 2023) and the ability to counteract the accumulation of Amyloid-beta in Alzheimer’s disease, Fenchol (Razazan et al., 2021)

To the best of our knowledge, this is the first report applying a metabolomics approach to investigate *C. sativa* polyploids in their diploid, triploid and tetraploid status across different cultivars. Overall, the response to polyploidization on morphological traits aligns with the observations on other plant species, showing larger organs with increasing ploidy levels. Moreover, analysis of secondary metabolites revealed for cannabinoids a marked decrease across cultivars in the higher ploidy levels. On the other hand, volatiles were negatively affected in triploids, while a differential response based on the cultivars’ genetic background was observed in tetraploids.

Our observations revealed that the effect of gene doubling appears to be more evident on minor compounds, making polyploidy a very promising tool to increase their concentrations. *C. sativa* presents at least 150 minor cannabinoids apart from THCA and CBDA (Hanuš et al., 2016) and many other secondary metabolites (Radwan et al., 2021) with medicinal potential (Pollastro et al., 2018) that were not considered in this study and could be affected in the tetraploid state. Further research on *C. sativa* tetraploids applying metabolomic approaches could reveal changes also in minor cannabinoids and other metabolite classes with potential biological activity and medicinal properties. Research on preclinical and animal models pointed out that *C. sativa* botanical preparations appear more effective than pure THC (Blasco-Benito et al., 2018) or CBD (Gallily et al., 2015). Yet it is not clear what compounds or combination of compounds are responsible for such increased activity. Given the shift observed in secondary metabolite quantity and diversity, follow-up tests on tetraploid *C. sativa* botanical preparations could be a first step to reveal what kind of changes polyploidization bring about in the plant’s pharmacological potential. Moreover, the only CBDA dominant cultivar of the study showed the most severe changes in cannabinoid and volatile reduction in the higher ploidy levels. Follow-up studies with more cultivars might elucidate what kind of relationship, if present, there might be between the effects of polyploidization and the genetic background of the plants.

The research on *C. sativa* polyploids is still in its infancy, and with this study, we have just scratched the surface. More research applying genomics, transcriptomics, metabolomics and phytocannabinomics (Cerrato et al., 2021) could reveal significant changes induced by genome doubling and elucidate mechanisms by which secondary metabolites are influenced in *C. sativa* polyploids.

Overall, this study found that polyploidization of *C. sativa* is a suitable approach to improve its medicinal potential, while the response is cultivar and genotype-dependent. The findings and methodology of this research lay the ground for further improving, evaluating and harnessing *C. sativa* chemical diversity by the breeding, biotechnological and pharmaceutical sectors.

## Data availability statement

The raw data supporting the conclusions of this article will be made available by the authors, without undue reservation.

## Author contributions

HPF: Headspace analysis, data acquisition, multivariate analysis, manuscript writing, review & editing. YHC: Supervising chemical analysis & manuscript review. KV: Conceptualization, project administration, formal analysis, supervision, manuscript writing, review & editing. MB: Conceptualization. BS: Plant growth, cannabinoid analysis, ploidy analysis, data acquisition, statistical analysis, formal analysis, manuscript writing, review & editing. FT: Conceptualization, supervision, methodology, plant growth, ploidy analysis, cannabinoid analysis, data acquisition, statistical analysis, formal analysis, investigation, manuscript writing, review & editing. All authors contributed to the article and approved the submitted version.

## Supplementary material

Supplementary Figure 1 The picture shows a side branch of a diploid (left side) and tetraploid (right side) genotypes of cultivar A.

Supplementary Figure 2 Principal component analysis PC1 against PC2, using UV scaling preprocessing data, of 4 *Cannabis sativa* L. cultivars A–D **(A)** and three ploidy levels 2n, 3n and 4n **(B)**. The PCA is based on 248 identified compounds. The explained variation is R^2^X= 0.28.
