# Supplementary figures and images for: Cultivar-dependent phenotypic and chemotypic responses of drug-type Cannabis sativa L. to polyploidization
Source: Front Plant Sci. 2023 Aug 11;14:1233191. doi: 10.3389/fpls.2023.1233191 (PMC10455935; doi:10.3389/fpls.2023.1233191)

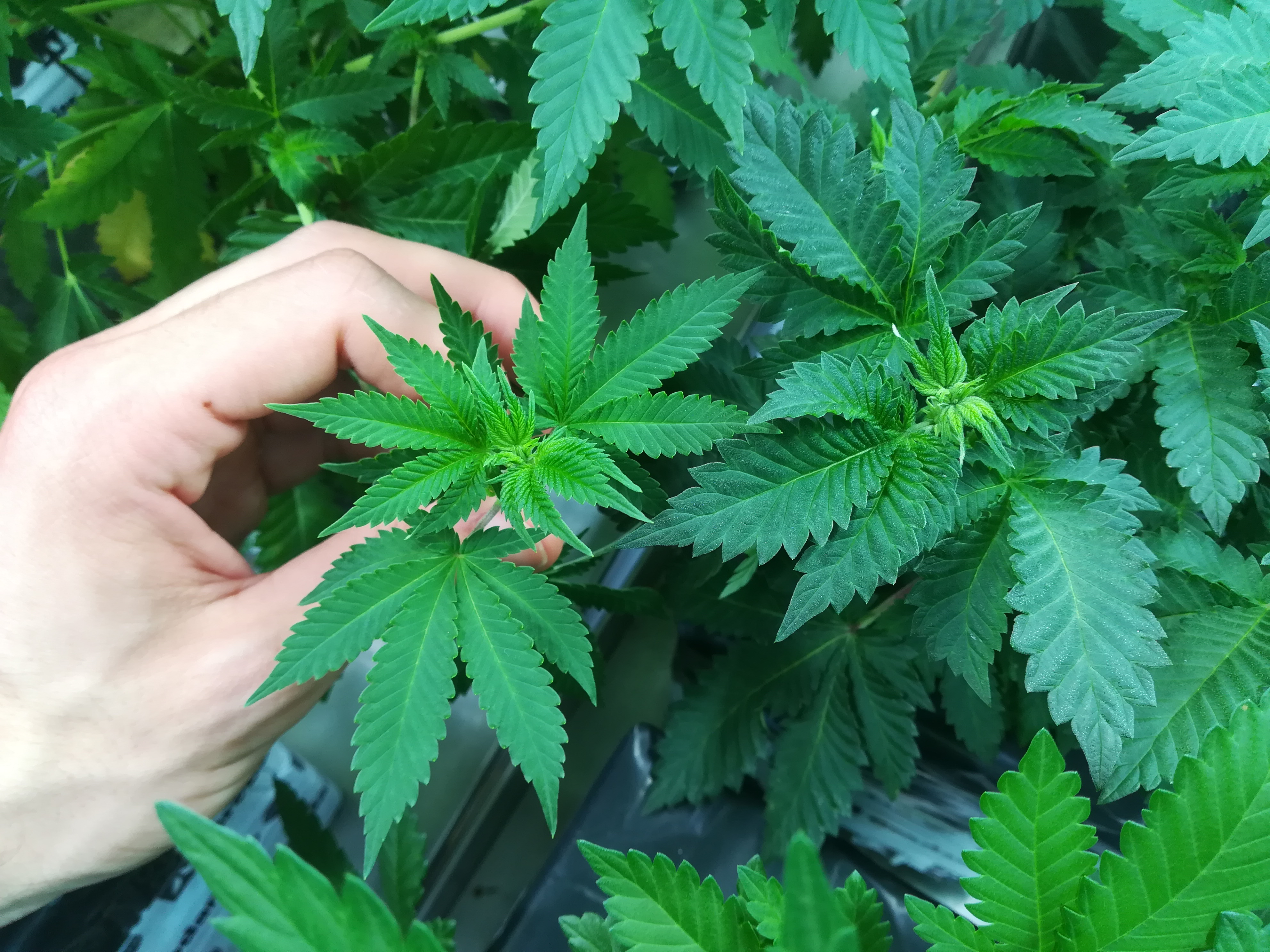

Supplement: Supplementary Figure 1 — The picture shows a side branch of a diploid (left side) and tetraploid (right side) genotypes of cultivar A. [file Image_1.jpeg]

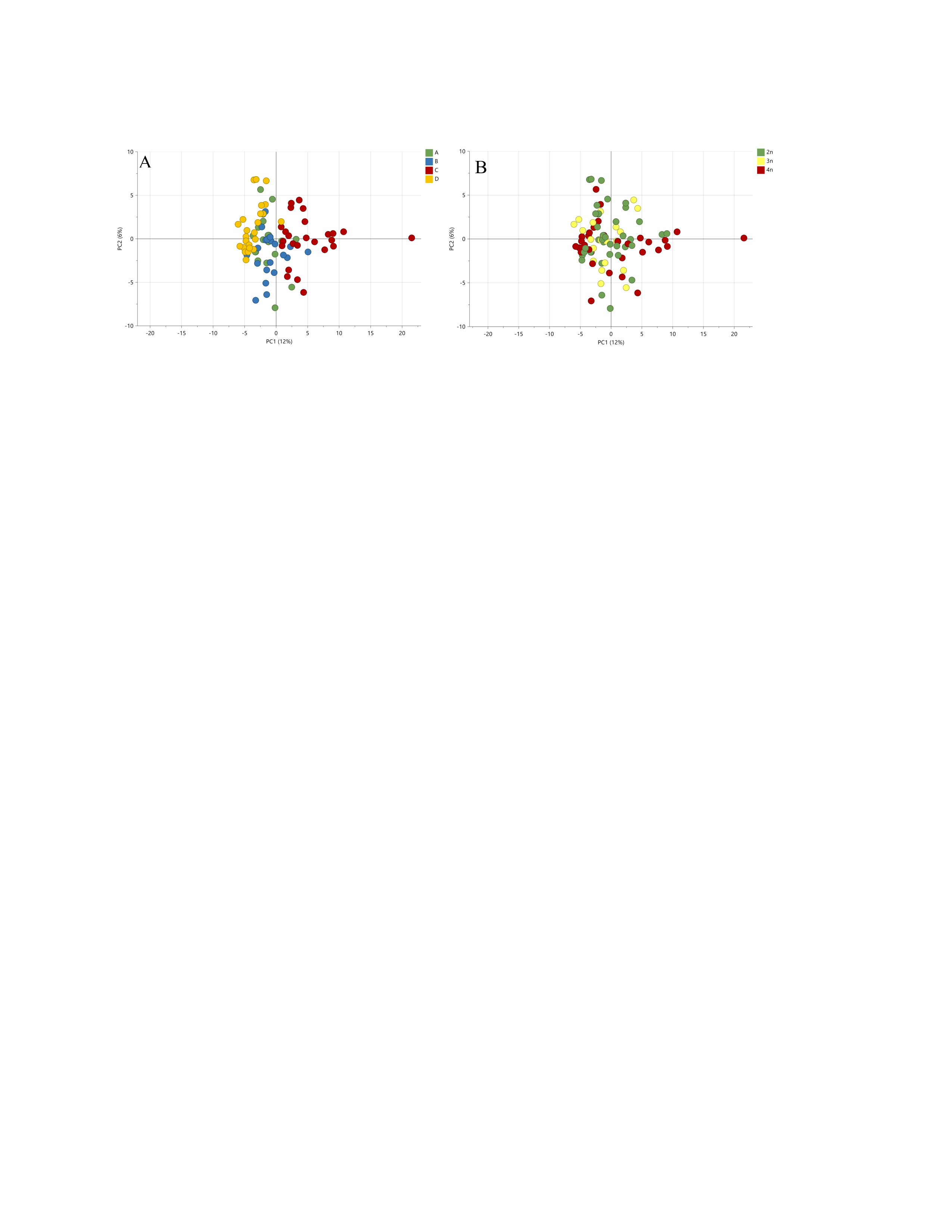

Supplement: Supplementary Figure 2 — Principal component analysis PC1 against PC2, using UV scaling preprocessing data, of 4 Cannabis sativa L. cultivars A–D (A) and three ploidy levels 2n, 3n and 4n (B). The PCA is based on 248 identified compounds. The explained variation is R2X= 0.28. [file Image_2.jpeg]
